# Optimization of Enzyme Essays to Enhance Reliability of Activity Measurements in Leukocyte Lysates for the Diagnosis of Metachromatic Leukodystrophy and Gangliosidoses

**DOI:** 10.3390/cells9122553

**Published:** 2020-11-28

**Authors:** Sebastian Strobel, Naomi Hesse, Vidiyaah Santhanakumaran, Samuel Groeschel, Gernot Bruchelt, Ingeborg Krägeloh-Mann, Judith Böhringer

**Affiliations:** Department of Pediatric Neurology, University Children’s Hospital Tübingen, Hoppe-Seyler-Straße 1, 72076 Tübingen, Germany; sestrob@gmail.com (S.S.); samuel.groeschel@med.uni-tuebingen.de (S.G.); gernot.bruchelt@web.de (G.B.); ingeborg.kraegeloh-mann@med.uni-tuebingen.de (I.K.-M.); Judith.Boehringer@gmx.de (J.B.)

**Keywords:** lysosomal storage disease, sphingolipidoses, metachromatic leukodystrophy, gangliosidoses

## Abstract

(1) Lysosomal storage diseases are rare inherited disorders with no standardized or commercially available tests for biochemical diagnosis. We present factors influencing the quality of enzyme assays for metachromatic leukodystrophy (MLD) and gangliosidoses (GM1; GM2 variants B and 0) and validate the reliability and stability of testing in a retrospective analysis of 725 samples. (2) Patient leukocytes were isolated from ethylene-diamine-tetra-acetic acid (EDTA) blood and separated for subpopulation experiments using density gradient centrifugation or magnetic cell separation. Enzyme activities in whole leukocyte lysate and leukocyte subpopulations were determined. (3) The enzyme activities in leukocyte subpopulations differed significantly. Compared to lymphocytes, the respective enzyme activities were 2.31–4.57-fold higher in monocytes and 1.64–2.81-fold higher in granulocytes. During sample preparation, a considerable amount of the lysosomal enzymes was released from granulocytes. Nevertheless, with the sample preparation method used here, total leukocyte count proved to be more accurate than total protein amount as a reference unit for enzyme activities. Subsequent analysis of 725 individuals showed clear discrimination of enzyme activities in patient samples (48 MLD; 21 gangliosidoses), with a sensitivity of 100% and specificity of 98–99%.

## 1. Introduction

Sphingolipidoses are a subgroup of lysosomal storage disorders [1], in which deficiency of a specific lysosomal enzyme leads to intralysosomal accumulation of the respective sphingolipid. This disease group is often associated with central nervous system involvement and usually a rapid progression with early death in early phenotypes and a rather variable disease course for juvenile and adult forms.

Sphingolipidoses are among the rare diseases [1]. This rareness results in a lack of knowledge about these diseases, often leading to a late diagnosis [2], which can have dramatic consequences, especially in the light of upcoming therapeutic possibilities that rely on early diagnosis [3,4,5,6]. In addition to genetic testing, biochemical analysis, which primarily involves measuring enzyme activities, plays a decisive role in diagnosing these disorders. As genotype–phenotype correlation is limited, the amount of residual enzyme activity may help in predicting the course of the disease as has been shown, for example, in metachromatic leukodystrophy (MLD) [7]. However, due to the rareness of lysosomal storage disorders, there are no standardized tests available and only a small number of specialized laboratories focus on their diagnosis. Thus, internal quality controls and external European-wide proficiency testing [8] are carried out to ensure reliable diagnosis. Nevertheless, determination of lysosomal storage diseases is a challenge in routine diagnostics.

In patients with suspected sphingolipidoses, leukocytes or skin fibroblasts are routinely used for enzyme activity measurements [9,10,11,12,13]. Leukocytes from peripheral blood are the preferred material especially due to easy sample collection [12]. After leukocyte isolation, lysates of unseparated leukocytes are mostly used for enzyme analysis. However, it is known that leukocyte subpopulations (granulocytes, monocytes, lymphocytes) harbor different concentrations and specific activities of lysosomal enzymes [14,15,16]. Therefore, our first aim was to investigate the enzyme activities of arylsulfatase A (ARSA, indicative for MLD) [17], β-galactosidase (β-Gal, indicative for GM1-gangliosidosis), β-hexosaminidase A (HEX A, indicative for GM2-gangliosidosis variant B (Tay Sachs disease)) and β-hexosaminidase A and B (HEX A+B, indicative for GM2-Gangliosidosis variant 0 (Sandhoff disease)) [18,19] in separated leukocyte fractions. Subsequently, we evaluated the importance of leukocyte composition on the measured enzyme activity when using unseparated leukocyte samples for diagnosis. Furthermore, time for transportation (gap between blood sampling and leukocyte isolation) and sample processing leads to cell stress, to which granulocytes react with degranulation measured by the release of myeloperoxidase, the marker enzyme for blood cell analysis on the ADVIA 120 cell counter [20,21,22]. Therefore, we asked whether the four lysosomal enzymes investigated in our study are also partially released due to degranulation of granulocytes during leukocyte isolation and discussed which reference system (total protein amount or number of leukocytes) seems appropriate. Finally, statistical assay validation was performed by retrospectively reanalyzing the enzyme activities in 725 samples that had been measured over 7 years in our laboratory.

## 2. Materials and Methods

### 2.1. Separation of Leukocyte Subpopulations and Degranulation Experiments

#### 2.1.1. Isolation of Granulocytes and Mononuclear Blood Cells

Briefly, 15 mL Histopaque 1119 (Merck, Darmstadt, Germany) was overlaid with 15 mL Histopaque 1077 solution. Then, 20 mL of heparinized blood (100 I.U. sodium heparin/mL, Roth, Karlsruhe, Germany) was carefully overlaid, followed by centrifugation at 630× *g* without braking at 18 °C for 25 min. The mononuclear cell fraction located between the plasma and Histopaque 1077, and the second layer containing granulocytes were collected. After washing, and resuspension of cell fractions in phosphate-buffered saline (PBS without Ca++/Mg++; glucose (1 g/L; 5.5 mM), Merck, Darmstadt, Germany), cell number was determined using an ADVIA-120 blood cell counter (Siemens, München, Germany). One part of the granulocyte suspension was used for degranulation experiments (see Section 2.1.3), another part was used for direct measurement of enzyme activities.

#### 2.1.2. MiniMACS Separation of Lymphocytes and Monocytes from Mononuclear Cells

Monocytes were separated using Magnetic Cell Sorting technology (MACS). The mononuclear cell fraction was incubated with an iron-particle-bound antibody cocktail provided by the manufacturer (CD3, CD7, CD19, CD45RA, CD56 and anti-IgE antibodies, Miltenyi Biotec, Bergisch Gladbach, Germany) according to the instructions of the company. Isolated cells were differentiated microscopically after cytospin preparation and Pappenheim staining.

#### 2.1.3. Degranulation Experiments Using Isolated Granulocytes

Isolated granulocytes were divided into three fractions (each 500 µL). The first fraction was used without further additives and showed baseline degranulation. The second fraction was incubated for 15 min at 37 °C with 10 µL cytochalasin B (250 µg/mL, Merck, Darmstadt, Germany) and 10 µL N-formyl-L-methionyl-L-leucyl-L-phenylalanine (N-fMLP, 5 × 10^−6^ mol/L, Merck, Darmstadt, Germany) for a moderate additional degranulation. In the third fraction, maximum degranulation was induced by additional incubation with granulocyte macrophage colony-stimulating factor (GM-CSF) (500 U, Amgen, Thousand Oaks, CA, USA) for 2 h at 37 °C. Enzyme activities were estimated in the supernatants of the granulocyte suspensions.

#### 2.1.4. Measurement of Myeloperoxidase in Supernatants of Granulocytes after Degranulation

The myeloperoxidase (MPO) analysis was carried out with an MPO enzyme-linked immunosorbent assay (ELISA) KIT (Merck, Darmstadt, Germany), using 100 µL supernatant or cell lysate according to the manufacturer’s instructions.

### 2.2. Leukocyte Preparations and Blood Cell Analysis

Blood counts were generated for ethylene-diamine-tetra-acetic acid (EDTA) blood samples with an ADVIA-120 cell counter once before sample preparation in whole blood and once after leukocyte isolation. To separate the leukocyte fraction from erythrocytes, dextran solution (5 g dextran 250 (Roth, Karlsruhe, Germany), 0.7 g sodium chloride (Merck, Darmstadt, Germany) and 50 mg heparin sodium salt (Roth, Karlsruhe, Germany) in 100 mL distilled water) was added to EDTA blood at a 1:5 ratio. Samples were mixed and stored for 1 h at room temperature for sedimentation of erythrocytes. The cell count of the leukocyte-containing supernatant was determined and after centrifugation, the cell pellet was resuspended in 500 µL distilled water for 5–10 × 10^6^ cells and stored at −20 °C until analysis. The enzyme activities were normalized to the total leukocyte number measured in the isolated sample (see Section 2.3).

### 2.3. Enzyme Assays of Sphingolipidoses

All four photometric enzyme assays were performed with established artificial substrates (Merck, Darmstadt, Germany). All reactions were stopped with 250 µL 0.5 M NaOH before the absorbance was measured. Blanks were measured by using water instead of sample. The sample background was determined by addition of 250 µL 0.5 M NaOH before incubation. Measured “sample background” values were used to subtract the background color from the measured enzyme activities of each sample. Test results are given as the Absorbance (E) of the formed products/10^6^ leukocytes or as nmol/h/10^6^ leukocytes. Minimal permissible values were determined internally by analyzing pathological and control cohorts (see Section 3.3.3).

#### 2.3.1. Arylsulfatase A

Para-nitrocatecholsulfate (pNCS) was used to determine the ARSA activity, which catalyzes the reaction to p-nitrocatechol and sulfate. The test was carried out according to Lee-Vaupel and Conzelmann [23] with variations described by Böhringer et al. [24]. In brief: 50 µL cell lysate was mixed with 200 µL of the substrate solution (10 mM pNCS, 0.5 mM sodium pyrophosphate and 10% sodium chloride in 0.5 M sodium acetate buffer, pH 5.0) and incubated for 48 h at 8 °C. Absorbance was measured at 514 nm on a spectrophotometer (Beckmann Coulter, Brea, CA, USA).

#### 2.3.2. β-Galactosidase

P-nitrophenyl-β-d-galactopyranoside was used to determine the β-Gal activity, which catalyzes the reaction to p-nitrophenol and β-d-galactopyranoside; 40 µL cell lysate was incubated with 150 µL substrate solution (2 mM p-nitrophenyl-β-d-galactopyranoside in 0.25 M sodium acetate, and 0.5 M sodium chloride buffer, pH 4.5) and 100 µL acetate buffer (0.25 M sodium acetate buffer, pH 4.5) for 2 h at 37 °C. Absorbance was measured at 405 nm.

#### 2.3.3. β-Hexosaminidase A

P-nitrophenyl-6-sulfo-2-acetamido-2-deoxy-d-glucopyranoside was used to determine the HEX A activity, which catalyzes the reaction to p-nitrophenol and 6-sulfo-2-acetamido-2-deoxy-d-glucopyranoside; 30 µL leukocyte lysate was mixed with 225 µL citrate buffer (0.1 M citric acid, 0.2 M sodium phosphate dibasic dehydrate buffer, pH 4.0) and 75 µL substrate solution (175 mM p-nitrophenyl-6-sulfo-2-acetamino-2deoxy-y-d-glucopyranoside in Aqua Bidest water) and incubated for 100 min at 37 °C. Absorbance was measured at 405 nm.

#### 2.3.4. β-Hexosaminidase A+B

P-nitrophenyl-N-acetyl-β-d-glucoseaminide was used to determine whole β-hexosaminidase activity, which catalyzes the reaction to p-nitrophenol and N-acetyl-β-d-glucoseaminide; 30 µL cell lysate was incubated with 50 µL 0.1 M citrate buffer, pH 4.5 and 250 µL substrate solution (2 mM p-nitrophenyl N-acetyl-β-d-glucosaminide in 0.1 M citrate buffer) for 120 min at 37 °C. Absorbance was measured at 405 nm.

### 2.4. Statistical Analyses

Statistical analyses were conducted using SPSS software v25 (IBM Corp., Armonk, N.Y., USA). Specific tests are given in the respective paragraphs.

## 3. Results

### 3.1. Leukocytes as Enzyme Source—Potential Pitfalls

#### 3.1.1. Different Leukocytes Contain Different Lysosomal Enzymes Activities

First, isolated granulocytes, lymphocytes and monocytes were analyzed microscopically. Granulocytes showed a purity of 96–98%, and lymphocytes showed a purity of 90% ± 3%, contaminated with 6% ± 2% monocytes and 4% ± 2% granulocytes. Monocyte fractions were 81 ± 5% pure, contaminated with 3% ± 4% lymphocytes and 16% ± 6% granulocytes; *n* = 5 blood donors.

The activities of the analyzed lysosomal enzymes found in granulocytes and monocytes normalized to the activities measured in lymphocytes, as the fraction with the lowest activity (set as 1.0), are presented in Table 1. Monocyte lysates showed 2.31- to 4.57-fold higher enzyme activities than lymphocytes, and granulocyte lysates showed 1.64- to 2.81-fold higher activities.

#### 3.1.2. Loss of MPO and Other Lysosomal Enzymes in Granulocytes during Leukocyte Preparation

**Figure 1:**
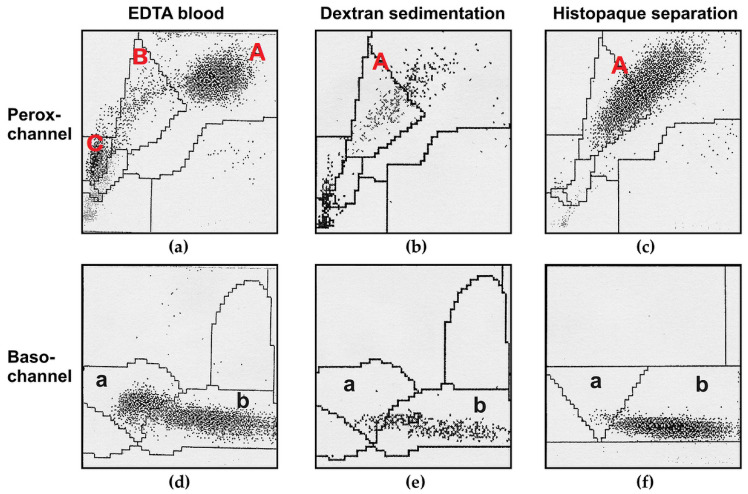
Presentation of blood cells on the ADVIA-120 blood cell counter before and after preparation of leukocytes via dextran or Histopaque separation. (**a**–**c**) Perox-channel: *x*-axis, myeloperoxidase (MPO) activity; *y*-axis, side scatter. (**d**–**f**) Baso-channel: *x*-axis, chromatin density (high angle signal 5–15°); *y*-axis, cell volume (low angle signal). (**a**,**d**) Normal cell distribution in whole blood measured in the perox-channel (A: neutrophil granulocytes, B: monocytes, C: lymphocytes; the small number of eosinophilic granulocytes are located in the area below A) and in the baso-channel (a: lymphocytes and monocytes, b: neutrophilic and eosinophilic granulocytes). (**b**,**e**) Distribution of cell populations after dextran sedimentation. The neutrophil granulocyte population in the perox-channel shifted partly into the monocyte gate due to MPO release, whereas the population of granulocytes (neutrophilic and eosinophilic granulocytes) was unchanged in the baso-channel (measuring nucleus characteristics). (**c**,**f**) Distribution of separated granulocyte fraction after Histopaque isolation (>96% purity). The granulocyte fraction in the perox-channel showed a shift into the monocyte gate similar to that after dextran sedimentation. The population in the baso-channel was not influenced.

Lysosomal enzyme activities measured in isolated granulocytes after dextran sedimentation are significantly lower than in whole blood because of their high fragility and sensitivity to mechanical stress during isolation, leading to degranulation and a partial release of lysosomal enzymes from storage vesicles [21,22]. This can be demonstrated using the ADVIA-120 system [25] which differentiates leukocytes by their intracellular myeloperoxidase (MPO) activity (in the perox-channel) and the nucleus shape (in the baso-channel) (Figure 1).

The neutrophil granulocyte population in whole blood samples shifted to the left after dextran or Histopaque separation in the Perox-channel (population A in the perox-channel; Figure 1a–c) but appeared at the same place in the baso-channel (population b in the baso-channel; Figure 1d–f), indicating that a considerable amount of MPO from the lysosomal storage vesicles of granulocytes was released during preparation (Figure 1).

**Figure 2:**
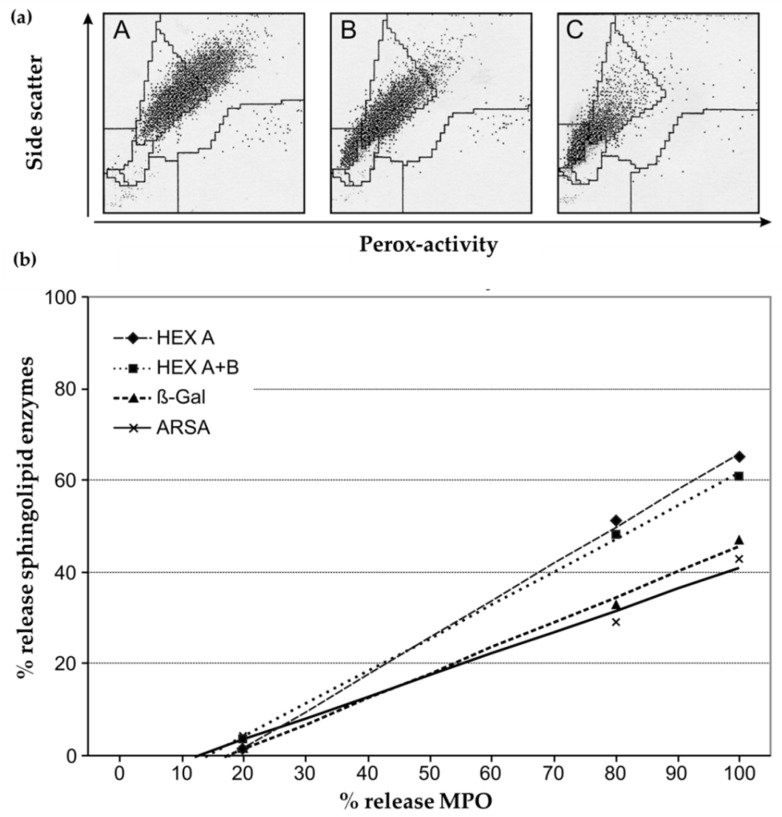
Correlation between release of MPO and other lysosomal enzymes from neutrophilic granulocytes into the supernatant: (**a**) Localization of the neutrophilic population (ADVIA 120, Histopaque separation) after different degranulation conditions: A: incubation without any additives; B: incubation with cytochalasin B + N-fMLP; C: incubation with granulocyte macrophage colony-stimulating factor (GM-CSF) + cytochalasin B + N-fMLP; (**b**) correlation between MPO (myeloperoxidase) amount (ELISA) and the activities of arylsulfatase A (ARSA), β-hexosaminidase A (HEX A), β-hexosaminidase A+B (HEX A+B) and β-galactosidase (β-Gal) in the supernatant of granulocyte suspensions after degranulation conditions as in the upper graph.

To compare MPO release and the release of other lysosomal enzymes in isolated granulocytes, MPO content in the supernatant was measured (ELISA) and compared with the release of ARSA, β-Gal, HEX A and HEX A+B under the same incubation conditions (Figure 2).

The release of lysosomal enzymes of isolated granulocytes could be enhanced by preincubation with cytochalasin B+N-fMLP and, most effectively, in the additional presence of GM-CSF (Figure 2a; see Section 2.1.3). The increase in MPO content in the supernatant correlated well with higher activities of all four of the measured lysosomal enzymes measured in the supernatant (Figure 2b), indicating that the amount of the lysosomal enzymes HEX A, HEX A+B, β-Gal and ARSA in isolated granulocytes is considerably lower than that in whole blood samples due to degranulation caused by mechanical stress during sample preparation.

### 3.2. Choice of a Suitable Reference System

In our laboratory, the enzyme activities are indicated as E (measured absorbance)/10^6^ leukocytes, whereby based on the respective calibration curves these values can easily be converted to nmol/h/10^6^ leukocytes. Thus, for the ARSA activity in the MLD assay, E = 0.1 corresponds to 3.8 nmoles p-nitrocatechol. For GM1-β-Gal activity E = 0.1 corresponds to 3 nmoles p-nitrophenol, and E = 0.1 corresponds to 3.6 nmoles p-nitrophenol in the assays for HEX A and HEX A+B, whereby the p-nitrophenol concentration varies between β-Gal, HEX A and HEX A+B assays due to the individual buffer systems, different volumes and general differences in the assay conditions for each of these lysosomal enzymes.

In the external quality studies by the European Research Network for Evaluation and Improvement of Screening, Diagnosis and Treatment of inherited Disorders of Metabolism (ERNDIM) with pure fibroblast lysates, enzyme activity results must be presented as nmoles product generated/hour/mg cellular protein amount, which makes sense for homogenous samples. As we are using inhomogeneous leukocyte lysates as the enzyme source, we reconsidered whether the protein amount is a proper reference unit. When using dextran sedimentation for leukocyte isolation, more than 99% of erythrocytes can be removed from the sample: the rate of erythrocytes to leukocytes in EDTA blood samples investigated in our study was 834 ± 79:1 (mean ± standard deviation, min–max: 634–977, *n* = 330). (Normal range: ca. 4–6 × 10^9^ erythrocytes/mL; 4–10 × 10^6^/mL leukocytes). This ratio was drastically reduced to 5.64 ± 1.23: 1 (min–max: 3.52–7.97; *n* = 330) after the sample preparation process. However, in the leukocyte lysate used for enzyme activity measurements the erythrocyte contamination is still approximately five times higher than the number of leukocytes.

The mean protein amount in erythrocytes corresponds to the mean hemoglobin (Hb) concentration and is approximately 26–34 µg/10^6^ cells [26]. Compared to this, the mean protein concentration in total leukocytes in a normally distributed blood count is rather similar (granulocytes: ca. 50 µg/10^6^ cells; lymphocytes: 10–15 µg/10^6^ cells (measured with bovine serum albumin as the protein standard)). Thus, it appears that, when measuring the total protein amount in leukocyte lysates, where there is a still high contamination with erythrocytes, the majority of protein measured stems from erythrocytes. Furthermore, this contamination is variable in every prepared sample, leading to additional variations in measurements normalized to the total protein amount. Contaminating erythrocytes could be subsequently eliminated by treatment with NH_4_Cl solution [27], but this procedure requires additional centrifugation steps that cause more degranulation and thereby might influence the quality of the leukocytes and follow lysosomal enzyme activity measurements (see Section 3.1).

When measuring the blood count in the total leukocyte lysate after sample preparation and using total leukocyte number as a reference unit, it is possible to relate the measured enzyme activity to the actual “source of enzyme activity”, namely the leukocytes. However, being aware of the contaminating erythrocytes, we investigated whether it is possible in practice to normalize enzyme activities to the leukocyte number ensuring results that allow a clear discrimination between normal and pathological samples. In the following part of the study, we were able to show that relating the measured enzyme activities to the total number of leukocytes with normal leukocyte distribution (41–75% granulocytes; 20–45% lymphocytes; 2–8% monocytes [26]) allowed unambiguous discrimination between healthy donors and patients with the respective enzyme defect (see Section 3.3.3). Only in some rare cases with unusually high numbers of lymphocytes or in leukemia samples were the measured activities below the minimal permissible value and led to suspected false pathological values. This could easily be corrected by looking at the patient’s differential leukocyte count (high number of lymphocytes, the fraction with the lowest enzyme activities).

### 3.3. Statistical Evaluation during a Seven-Year Observation Period

#### 3.3.1. Inter-assay Variability (Internal Control Person)

To determine the consistency of measurements and inter-assay variability, the enzyme activity in a healthy individual (female, age 57 years at start of measurements) was measured over 7 years within every analytical run. ARSA enzyme activity was 1.75 ± 0.13 E_514nm_/10^6^ leukocytes (1.39 ± 0.1 nmol/h/10^6^ leukocytes; (*n* = 331)), the β-Gal activity was 2.05 ± 0.16 E_405nm_/10^6^ leukocytes (30.75 ± 2.4 nmol/h/10^6^ leukocytes; (*n* = 326)), HEX A activity was 0.96 ± 0.05 E_405nm_/10^6^ leukocytes (20.73 ± 1.07 nmol/h/10^6^ leukocytes; (*n* = 327)) and HEX A+B activity was 4.61 ± 0.43 E_405nm_/10^6^ leukocytes (83 ± 7.74 nmol/h/10^6^ leukocytes; (*n* = 330)). Thus, the standard deviation of all four enzyme activities over 7 years was less than 10%.

#### 3.3.2. Demographic Characterization of the Cohort

Data collected over a period of 7 years in the neurometabolic laboratory of the children’s University Hospital Tübingen were reanalyzed regarding activities of the enzymes ARSA, β-Gal, HEX A and HEX A+B. In total, 725 individuals were tested within this timeframe (50.2% female, 47.7% male and 2.1% were of unknown gender). Individuals without pathological enzyme activity were defined as the normal cohort (*n* = 656; 29 ± 24 years at the time of testing) and individuals diagnosed with MLD, GM1 or GM2, and thus showing deficiency for the respective enzyme tested, were defined as the pathological cohort (in total *n* = 69), from which 48 MLD patients, 3 GM1 gangliosidosis patients, 14 patients with Tay–Sachs disease (GM2 gangliosidosis variant B), and 4 patients with Sandhoff disease (GM2 gangliosidosis variant 0) were identified (Table 2).

#### 3.3.3. Enzyme Activity in the Normal Cohort and Minimal Permissible Value

**Figure 3:**
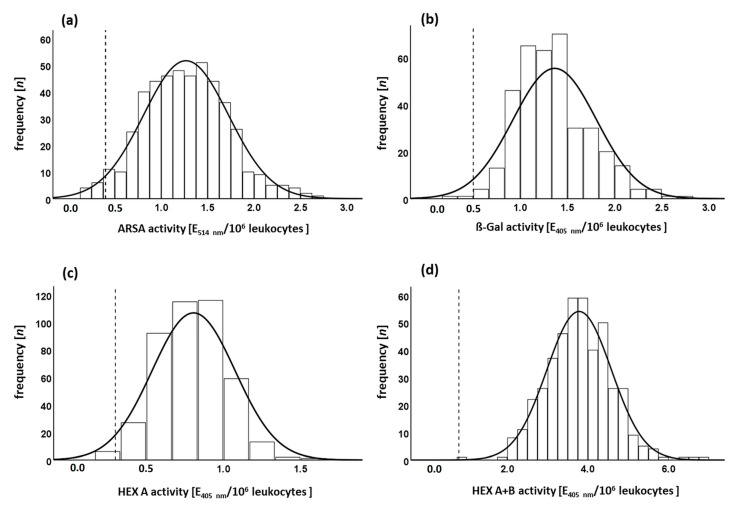
Enzyme activities in normal cohorts: Frequency distributions of enzyme activities measured in the normal cohort show a normal Gaussian distribution. Set minimal permissible values are indicated as dashed vertical lines. (**a**) The average arylsulfatase A activity (ARSA) was 1.27 ± 0.46 E_514nm_/10^6^ leukocytes (*n* = 474), and 2.3% of the samples were a false pathological with measured values below the minimal permissible value (0.4 E_514nm_/10^6^); (**b**) The average β-galactosidase activity (β-Gal) was 1.36 ± 0.44 E_405nm_/10^6^ leukocytes (*n* = 369), and 0.5% of the samples were false pathological with measured values below the minimal permissible value (0.5 E_405nm_/10^6^ leukocytes); (**c**) the average β-hexosaminidase A activity (HEX A) was 0.81 ± 0.27 E_405nm_/10^6^ leukocytes (*n* = 432), and 0.7% of the samples were a false pathological with measured values below the minimal permissible value 0.3 E_405nm_/10^6^ leukocytes); (**d**) the average β-hexosaminidase A and B activity (HEX A+B) was 3.78 ± 0.80 E_405nm_/10^6^ leukocytes (*n* = 434), and 0.2% of the samples were a false pathological with measured values below the minimal permissible value 0.8 E_405nm_/10^6^ leukocytes.

The normal cohort without enzyme deficiency (heterozygosity not excluded) showed normal Gaussian distribution for all four enzyme activities (Figure 3).

For differentiation between normal and pathological enzyme activities, minimal permissible values were carefully chosen with a significant clearance distance (Table 2 and Table 3).

For the ARSA activity measurements, the set minimal permissible value was 31% of the average enzyme activity of normal cohort. Therefore, 2.3% of the normal cohort (*n* = 474) showed a false pathological activity of less than 0.4 E_514nm_/10^6^ leukocytes (Figure 3a). For β-galactosidase activity (*n* = 369), the minimal permissible value was 36% of average of normal controls leading to 0.5% false pathological results (Figure 3b). For β-hexosaminidase A (*n* = 432) and β-hexosaminidase A and B (*n* = 434), minimal permissible values were set at 38% and 21% of average activity of normal control. The rate of false-positive results was 0.7% and 0.2%, respectively (Figure 3c,d). All minimal permissible values are intentionally higher than the theoretical thresholds of effective substrate turnover (10–15% enzyme activity [28]) to minimize the risk of false-negative results. False pathological results could be corrected before diagnosis by, e.g., repeating the enzyme activity measurement, looking at cell composition (compare Section 3.1) and considering clinical data.

##### Effects of Age and Transport Time on Enzyme Activity in the Normal Cohort

The age of the individuals at the time of investigation was correlated with activity in all four enzymes using linear regression and showed no statistical correlation (*p* > 0.05).

Transport time (gap between blood collection and leukocyte preparation) as a parameter influencing enzyme activity was analyzed with a scatter plot and linear regression (*n* = 523). As the most sensitive enzyme in this cohort, β-galactosidase showed a statistically significant reduction in enzyme activity of 0.04 E_405nm_/10^6^ leukocytes per day (*p* = 0.02). For all other enzymes, no statistically relevant influence of transport duration was observed on enzyme activity. The mean enzyme activity was relatively constant during transport within 4 days. Blood samples, that were stored at room temperature for longer than 4 days, were very poor for sample preparation because by then, hemolysis of the blood sample proved to be the limiting factor.

#### 3.3.4. Enzyme Activities in Pathological Cohorts

Table 2 shows a detailed characterization of the pathological cohorts. MLD patients showed residual ARSA activity of 1.6%, whereas the activities of all the other lysosomal enzymes measured in MLD patients were normal. On average, GM1-gangliosidosis patients showed β-Gal activities of 3.9% of the normal cohort and had normal activities of the other lysosomal enzymes. HEX A activity of affected individuals was 6.25% of the average normal cohort activity. Compared to the HEX A+B activity in the normal cohort, patients with Sandhoff disease showed 5.6–12.7% residual activity. HEX A activity of these patients showed also reduced activities of 0.11–0.26 E_405nm_/10^6^ leukocytes below the minimal permissible value (0.3) as expected but clearly above enzyme activity values of patients with Tay Sachs disease.

#### 3.3.5. Evaluation of Diagnostic Tests

**Figure 4:**
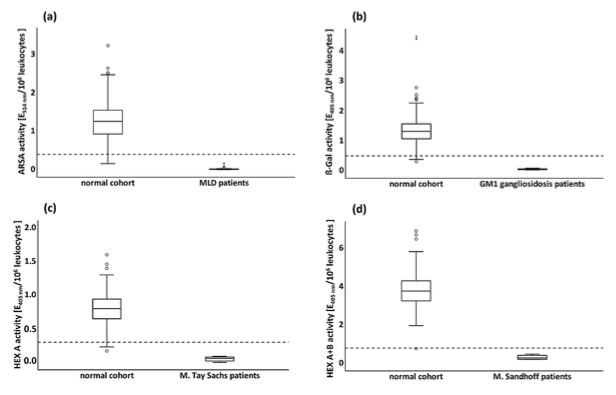
Boxplot of normal and pathological cohort for each analyzed lysosomal enzyme. Set minimal permissible values are indicated as dashed horizontal lines; ○: outliers. (**a**) Arylsulfatase A activity (ARSA) in normal (*n* = 474) and pathological cohort (*n* = 48); (**b**) β-galactosidase activity (β-Gal) in normal (*n* = 369) and pathological cohort (*n* = 3); (**c**) β-hexosaminidase A activity (HEX A) in normal (*n* = 432) and pathological cohort (*n* = 14); (**d**) β-hexosaminidase A and B activity (HEX A+B) in normal (*n* = 434) and pathological cohort (*n* = 4).

For comparison, the activities of the four analyzed enzymes in the pathological cohorts were plotted against those in the normal cohort with additional indication of the set minimal permissible values (Figure 4a–d).

The demarcation of the pathological cohorts from the minimal permissible value and above all from the healthy cohort is visually well recognizable. In this picture, it is clearly illustrated that the minimal permissible value was intentionally chosen very close to the healthy cohort, with high assay sensitivity as the main priority. Weaknesses in specificity can easily be compensated by considering all the clinical parameters.

**Figure 5:**
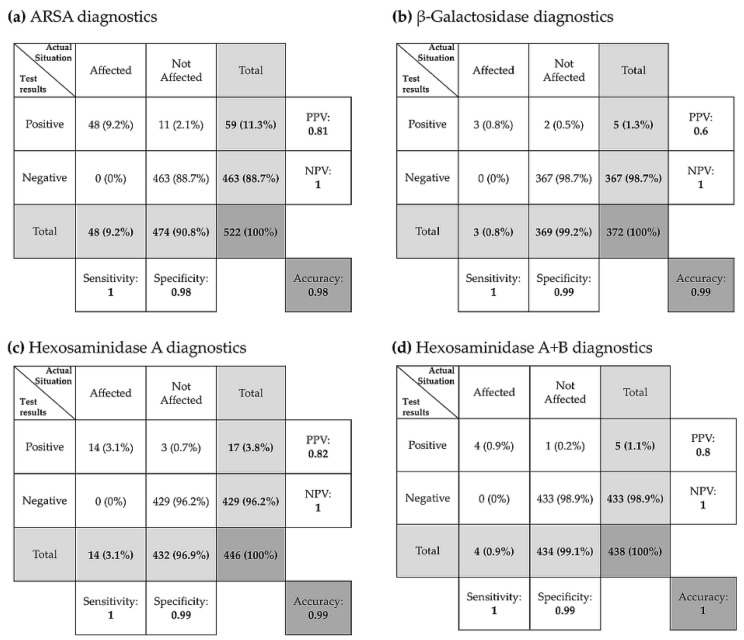
Contingency tables for ARSA, β-Gal, HEX A and HEX A+B diagnostics. With the help of contingency tables enzyme activity assays for arylsulfatase A (ARSA) (**a**), β-galactosidase (**b**), β-hexosaminidase A (**c**), and β-hexosaminidase A+B (**d**), were evaluated for their sensitivity, specificity, positive predictive value (PPV), negative predictive value (NPV) and accuracy.

With the help of contingency tables the four enzyme activity assays were evaluated for their sensitivity, specificity and accuracy (Figure 5).

Both the sensitivity and the negative predictive value were 100% in all tested enzyme activity assays, which was and is the highest priority in our routine laboratory. Because the minimal permissible value was intentionally chosen to be very high, the specificity and positive predictive value were slightly reduced (Figure 5). Overall, this resulted in an accuracy of 98% for the ARSA activity assay (Figure 5a), 99% for β-galactosidase activity assay (Figure 5b), 99% and 100% for the hexosaminidase activity assays (Figure 5c,d).

The most common cause of false-positive results was an abnormal blood count. Lymphocytes, e.g., have the lowest lysosomal enzyme activity among the leukocyte subpopulations. Thus, lymphocyte counts above the reference range, e.g., in leukemia patient samples (see Section 3.1), led to reduced measured total enzyme activity in the samples. Such abnormal blood counts due to high lymphocyte numbers were responsible for more than 50% of false-positive results in the analyzed cohort.

## 4. Discussion

This work shows that many factors play an essential role in the measurement of lysosomal enzyme activities and diagnosis of their deficiency. The composition of the blood sample is an important factor. Our results agree with results from the literature, showing that different leukocyte subpopulations have different lysosomal enzyme activity levels [14,15,16], with the lowest values in lymphocytes. The minimal permissive values chosen for discrimination between normal and pathological enzyme activities relate to blood samples with normal leukocyte distribution [26], indicating that the reference to the total number of leukocytes proved to be sufficient to clearly differentiate between healthy individuals and patients with the respective enzyme defect. If the number of lymphocytes is unusually high, e.g., in leukemia, suspected false pathological values can be measured. This can easily be corrected by considering the patient’s differential blood count. In addition, in contrast to a true pathological sample, all measured lysosomal enzyme values are also very low in such a situation.

Sample condition and the type of cell preparation also play a role. Transportation and preparation lead to cell stress, which granulocytes react to with MPO-degranulation [22]. Here, we confirmed that this degranulation also affects granules harboring the four lysosomal enzymes analyzed in this study. Due to degranulation, the released enzymes are no longer available for activity measurements within the cells and therefore the measured enzyme activities are lower than in whole blood samples. As this process equally affects samples from patients and healthy controls, it does not influence the test results. However, the reduced total enzyme concentration due to sample preparation reduces the sensitivity of the test. Thus, this information is important for the design of sample preparation, since one has to carefully weigh up the purity of the cell population (which means more intervention) and higher residual enzyme concentration in order to better distinguish between pathological and healthy samples.

Regarding sample condition the used anticoagulant during sample collection and the leukocyte isolation procedure are important factors, especially when using plasma as the enzyme source. EDTA, citrate and heparin are some of the commonly used anticoagulants with different properties depending on the analyzed lysosomal enzyme and the assay conditions [29,30,31]. However, when isolating cells from whole blood the majority of the anticoagulant is removed during the sample preparation process. In our laboratory, we prefer EDTA-blood, which is a routinely used sample type for analysis in many laboratories.

Furthermore, regarding leukocyte isolation and sample preparation we prefer the gentle dextran sedimentation method. Although removing up to 99% of the erythrocytes the remaining erythrocytes contamination is still approximately 5.6:1 in the leucocytes lysates. However, we chose this rather impure but gentle leukocyte isolation method to minimize additional enzyme release during sample preparation. A study on erythrocytes showed that activities of “lysosomal” hydrolases, e.g., β-galactosidase could be measured in these cells. Despite, the reported enzyme activity in erythrocytes being considerably lower than that of leukocytes [32] and a significant influence of the measured total enzyme activity seems unlikely; we minimized the risk of potential artifact measurements due to contaminating erythrocytes by intentionally setting the minimal permissible values clearly above the pathological range. In the statistical part of this study we were able to show that the risk of false-negative results was set to zero (see Section 3.3.5).

Finally, the reference system, to which the enzyme activities in leukocyte extracts are related to, is important. Due to the high and variable contamination with remaining erythrocytes after dextran sedimentation and the heterogeneity of a leukocyte sample itself, we use “cell number” as a reference unit and show that normalizing the enzyme activity to the total leukocyte number is an adequate alternative when using inhomogeneous samples such as leukocyte lysates as an enzyme source.

In addition to the enzyme activity test itself, the consistency of measurements over the years is also an important aspect. Therefore, we retrospectively evaluated our diagnostic tests over a period of 7 years. In total, 725 samples were investigated within this timeframe, in which 48 MLD, 3 GM1, 14 patients with Tay–Sachs disease and 4 patients with Sandhoff disease were identified. As shown in Section 3.3.1, the internal control values for all four enzymes were very stable and constant during this observation period. Different potential factors that could influence the results were analyzed; among them were patient age and the time between blood collection and isolation of leukocytes. The statistical analysis revealed no significant influence of the patients’ age on the enzyme activity in the analyzed cohort. Moreover, the mean enzyme activity was constant for up to 4 days between blood collection and preparation for 3 out of 4 tested enzymes, but hemolysis of the blood sample was the main limiting factor, which is consistent with the literature [29]. As expected, samples without enzyme deficiency show Gaussian distributions. The minimal permissible value was deliberately chosen to be very high (between 38% and 21% of the average activity in the normal control) to avoid false-negative results. The resulting risk of false-positive results was accepted because they can be identified by critical evaluation of sample composition and condition, clinical data and other additional parameters. Thus, based on our experience over the recent years, we could formally identify false-positive measurements before release of the results.

In summary, we show that, in addition to a robust, well-established enzyme assay, the enzyme source and its composition, cell preparation and the reference system play an important role in reliable diagnosis of lysosomal storage disorders.

## Figures and Tables

**Table 1:** Comparison of arylsulfatase A, β-hexosaminidase A, β-hexosaminidase A+B and β-galactosidase activities in the different leukocyte fractions normalized to lymphocyte fraction set as 1.0.

		Cell Fraction		
	*n* ^1^	Lymphocytes	Monocytes	Granulocytes
Hexosaminidase A+B	9	1.0	4.36 ± 1.05	1.81 ± 0.56
Hexosaminidase A	8	1.0	4.57 ± 1.59	2.81 ± 0.73
β-Galactosidase	8	1.0	4.47 ± 1.00	1.64 ± 0.41
Arylsulfatase A	9	1.0	2.31 ± 0.68	1.64 ± 0.38

^1^ (*n* = number of blood donors).

**Table 2:** Detailed characterization of the pathological cohort.

	MLD	GM1	Tay–Sachs Disease	Sandhoff Disease
**Number**	**48**	**3**	**14**	**4**
♂	24	2	8	2
♀	24	1	6	2
**Age at onset (years)**	**11.7 ± 12**	**7 ± 10**	**16 ± 17**	**0.9 ± 0.1**
Median	6.2	1.5	10	0.9
Min.	0.08	1.1	0.08	0.8
Max.	41.6	18.1	41	1
Minimal permissible value(E/10^6^ leukocyte)	0.4	0.5	0.3	0.8
**Average of measured enzyme activity ^1^** **(E/10^6^ leukocytes)**	**0.02 ± 0.03**	**0.05 ± 0.03**	**0.05 ± 0.03**	**0.32 ± 0.13**

^1^ depending on the disease minimal permissible value and measured enzyme activities of the respective affected enzyme is depicted: metachromatic leukodystrophy (MLD)—ARSA activity; GM1—β-Gal activity; Tay–Sachs disease—HEX A activity; Sandhoff disease—HEX A+B activity.

**Table 3:** Enzyme activity of normal cohort and minimal permissible value.

	ARSA	β-Gal	HEX A	HEX A+B
Number	474	369	432	434
Measured enzyme activity ^1^ (E/10^6^ leukocytes)	1.27 ± 0.46	1.36 ± 0.44	0.81 ± 0.27	3.78 ± 0.80
Minimal permissible value ^2^ (E/10^6^ leukocytes)	0.4	0.5	0.3	0.8

^1^ Measured enzyme activities converted to nmol/h/10^6^ leukocytes: ARSA: 1 ± 0.36 nmol p-nitrocatechol (ox)/h/10^6^ leukocytes (or 48.2 ± 17.5 nmol/48h/10^6^ leukocytes); β-Gal: 20.4 ± 6.6 nmol p-nitrophenol (ox)/h/10^6^ leukocytes; HEX A: 17.5 ± 5.83 nmol p-nitrophenol (ox)/h/10^6^ leukocytes; HEX A+B: 68 ± 14.4 nmol p-nitrophenol (ox)/h/10^6^ leukocytes; ^2^ minimal permissible values converted to nmol/h/10^6^ leukocytes: ARSA: 0.32; β-Gal: 7.5; HEX A: 6.48; HEX A+B: 14.4.
